# LANTERN 2: Association Between Gene Molecular Profile and STAS in Lung Adenocarcinoma: A Comparative Analysis in a Prospective Real-World Population

**DOI:** 10.3390/genes17060677

**Published:** 2026-06-09

**Authors:** Carolina Sassorossi, Davide Dalfovo, Elisa De Paolis, Jessica Evangelista, Alessandra Cancellieri, Annalisa Campanella, Luca Boldrini, Esther G. C. Troost, Róza Ádány, Núria Farré, Ece Öztürk, Angelo Minucci, Rocco Trisolini, Emilio Bria, Stefano Margaritora, Steffen Löck, Filippo Lococo

**Affiliations:** 1 Thoracic Surgery Unit, Catholic University of the Sacred Heart, 00168 Rome, Italy; annalisa.campanella@policlinicogemelli.it (A.C.);; 2 Thoracic Surgery Unit, A. Gemelli University Hospital Foundation IRCCS, 00168 Rome, Italy; 3 OncoRay—National Center for Radiation Research in Oncology, Faculty of Medicine and University Hospital Carl Gustav Carus, TUD Dresden University of Technology, Helmholtz-Zentrum Dresden-Rossendorf, 01062 Dresden, Germany; davide.dalfovo@tu-dresden.de (D.D.); esther.troost@uniklinikum-dresden.de (E.G.C.T.);; 4 Department of Radiotherapy and Radiation Oncology, Faculty of Medicine and University Hospital Carl Gustav Carus, TUD Dresden University of Technology, 01062 Dresden, Germany; 5 Departmental Unit of Molecular and Genomic Diagnostics, Genomics Core Facility, Gemelli Science and Technology Park (G-STeP), Fondazione Policlinico Universitario A. Gemelli IRCCS, 00168 Rome, Italy; elisa.depaolis@policlinicogemelli.it (E.D.P.); jessica.evangelista@policlinicogemelli.it (J.E.); angelo.minucci@policlinicogemelli.it (A.M.); 6 Clinical Chemistry, Biochemistry and Molecular Biology Operations (UOC), Fondazione Policlinico Universitario A. Gemelli IRCCS, 00168 Rome, Italy; 7 Pathology Unit, Department of Woman and Child’s Health and Public Health Sciences, Fondazione Policlinico Universitario Agostino Gemelli IRCCS, 00168 Rome, Italy; alessandra.cancellieri@policlinicogemelli.it; 8 Advanced Radiotherapy Center, A. Gemelli University Hospital Foundation IRCCS, 00168 Rome, Italy; luca.boldrini@policlinicogemelli.it; 9 Institute of Radiooncology—OncoRay, Helmholtz-Zentrum Dresden-Rossendorf, 01062 Dresden, Germany; 10 ELKH-DE Public Health Research Group, Department of Public Health and Epidemiology, Faculty of Medicine, University of Debrecen, 4032 Debrecen, Hungary; adany.roza@med.unideb.hu; 11 Institut de Recerca de Barcelona, Hospital de la Santa Creu i Sant Pau (IR-HSCSP), 08041 Barcelona, Spain; nfarre@santpau.cat; 12 School of Medicine, Turkey and Koç University Research Center for Translational Medicine (KUTTAM), Koç University, Sariyer, 34835 Istanbul, Turkey; ozturkece@ku.edu.tr; 13 Interventional Pulmonology Unit, A. Gemelli University Hospital Foundation IRCCS, 00168 Rome, Italy; 14 Medical Oncology, A. Gemelli University Hospital Foundation IRCCS, Largo A. Gemelli 8, 00168 Rome, Italy

**Keywords:** STAS, NSCLC, genomic alterations

## Abstract

**Introduction**: Lung cancer, the leading cause of cancer-related mortality worldwide, is a heterogeneous malignancy comprising distinct histological and molecular subtypes, with non-small cell lung cancer (NSCLC) accounting for approximately 85% of cases and adenocarcinoma (ADC) representing the most prevalent histotype. An emerging pathological feature of NSCLC, spread through air spaces (STAS)—defined as the extension of tumor cells into the lung parenchyma beyond the main tumor margin—has been associated with worse disease-free and overall survival and has been proposed as a possible predictor of recurrence to guide surgical extent. Concurrently, recent comprehensive genomic profiling of early-stage NSCLC has highlighted the need to interpret multi-omics data and their relationship with pathological variables, including IASLC histological subtypes, to better personalize treatment strategies. In this context, we investigated the overall distribution of STAS and its association with tumor mutational profiles and IASLC histological subtypes in a large real-world cohort of lung adenocarcinoma patients from the LANTERN project. **Materials and Methods**: In a prospective, multicenter observational study (March 2023–December 2024), 271 NSCLC patients were enrolled, and clinicopathological, immunohistochemical, and genomic data were collected; comprehensive genomic profiling was performed using the TruSight Oncology 500 assay to analyze 523 cancer-related genes, tumor mutational burden (TMB), and microsatellite instability; and STAS was assessed according to IASLC criteria. Adenocarcinoma accounted for roughly 90% of the cases, with a median age of 69 years and a predominance of stage IV disease (49.5%). STAS was evaluable in 162 cases and was detected in 17.9% of tumors. **Results**: STAS-positive tumors showed a higher trend towards locally advanced and advanced disease; no differences were observed in sex, age, smoking status, tumor mutational burden, or PD-L1 expression. Additionally, STAS-positive tumors showed a higher association with micropapillary, mucinous, and papillary patterns, whereas the acinar pattern was more frequent in STAS-negative tumors. The most frequently mutated genes were TP53, KRAS, EGFR, and STK11, with no significant differences between groups; ROS1 alterations were absent in STAS-negative tumors but detected more frequently in STAS-positive cases. **Conclusions**: Overall, these findings indicate that STAS positivity is associated with high-risk histological subtypes and advanced disease, suggesting its importance as a marker of tumor aggressiveness and emphasizing the need for its systematic evaluation in lung adenocarcinoma to better guide surgical planning and patient risk assessment.

## 1. Introduction

Lung cancer remains the leading cause of cancer death in the world, still presenting an overall dismal prognosis [1]. It remains a highly heterogeneous neoplasm in which various histological subgroups have been clearly identified, each with specific features and molecular characteristics [2]. Among non-small cell lung cancers (NSCLCs) (the most representative histology accounting for approximately 85% of all lung tumors), adenocarcinoma (ADC) represents the most frequent histotype (more than 60% of all NSCLCs) with specific histological patterns defined by IASLC/ATS/ERS and potentially associated with different prognostic outcomes [1].

Another pathological feature of NSCLC emerging in the last decade is the “Spread through air spaces” (STAS) that has been defined by Travis and coworkers in the current World Health Organization (WHO) Classification of Lung Tumors as the spread of micropapillary clusters, solid nests, and/or single cancer cells into air spaces in the lung parenchyma beyond the edge of the main tumor [3]. Although the definition of STAS varies across different studies, STAS has been shown to be associated with decreased disease-free survival and overall survival in multivariate analyses [4].

Regarding treatment, approximately 30% of patients with NSCLC are candidates for radical surgery (stages I–IIIA) and, in general, show a better prognosis than the advanced ones [5]. Sublobar resection (pulmonary parenchyma sparing surgery) for smaller peripheral nodules, instead of more extensive lobar resection, has become more common recently [6]. However, since the indications for performing sublobar resection are still a matter of large debate, there is an urgent need for identifying multiple predictive factors for recurrence that should be considered when choosing the best surgical resection option, especially pathological and molecular factors.

In this framework, some researchers have suggested that STAS could be considered to tailor treatment to more extensive lung resection (i.e., lobar resection), as it appears to act as an independent predictor of disease recurrence in patients undergoing sublobar resections for early-stage lung adenocarcinoma, despite the data available still being scarce and controversial [7].

On the other hand, a new grading system for pulmonary adenocarcinoma was recently proposed by IASLC/ATS/ERS [8], as reported above. The grading system is based on a combination of histological patterns, with an emphasis on high-grade patterns, which offers better prognostic correlation than the adenocarcinoma classification system, which is based solely on the predominant pattern.

Finally, there is an increasing interest in exploring the molecular landscape of genomic profiles even in early-stage NSCLC because it appears clear that every treatment (including surgery) should be personalized according to several tumor characteristics (i.e., biological aggressiveness). Indeed, in the last decade, the wide implementation of high-throughput technologies and comprehensive genomic profiling (CGP) in lung cancer allowed for the identification of a broad spectrum of molecular aberrations and altered signaling pathways, leading to the definition of distinct molecular profiles.

Thus, the interpretation of these complex data (mostly genomic and pathological variables) and their inter-relationship in NSCLC patients is pivotal for tailoring more precise therapeutic approaches.

Within this context, the LANTERN (Lung cancer multi-omics digital human avatars for integrating precision medicine) project [9] arises, with the aim of delivering a novel approach for comprehensive lung cancer decision-making solutions, based on predictive digital platforms powered by the integration of complex data.

In the present analysis, we analyze the LANTERN population, consisting of a large prospective cohort of Caucasian lung adenocarcinoma patients, with the following aims:

(1) To explore the overall expression of STAS and its distribution according to staging and other clinical factors;
(2) To analyze the inter-relationship between STAS positivity, tumor mutational profile, and IASLC morphological classification.

## 2. Materials and Methods

### 2.1. Ethics

The protocol of the study was designed in accordance with the Standards of Good Clinical Practice of the European Union and the current review of the Helsinki Declaration and was approved by the respective Ethics Committee. All methods were carried out in accordance with relevant guidelines and regulations. Informed consent was obtained from all subjects and/or their legal guardian(s). Approval of Fondazione Policlinico Universitario Agostino Gemelli IRCCS—Università Cattolica del Sacro Cuore Ethics Committee Number: 5420-0002485/23; Trial registration—clinicaltrial.gov: NCT05802771.

### 2.2. Patients

This study was conducted on a prospective, multicenter observational cohort of 271 patients with non-small cell lung cancer (NSCLC), enrolled between March 2023 and December 2024 in the LANTERN consortium (see Consort Diagram—Supplementary Figure S1) [9]. No restrictions regarding sex or disease stage were applied at enrollment in order to ensure a representative NSCLC cohort and minimize potential selection bias.

The inclusion criteria were as follows: (1) age ≥ 18 years; (2) provision of written informed consent; and (3) histopathological confirmation of NSCLC.

Patients were excluded in the presence of: (1) a diagnosis of neuroendocrine carcinoma, small cell lung carcinoma, or other non-NSCLC lung malignancies (see details below); and (2) a diagnosis based solely on cytological specimens.

Tumor staging was performed according to the eighth edition of the TNM classification established by the American Joint Committee on Cancer (AJCC) for lung cancer [10]. Clinicopathological variables were retrieved from electronic medical records, including age, sex, smoking status, tumor site, histopathological characteristics, presence of STAS, PD-L1 expression, treatment details, and pathological TNM stage.

Clinical and molecular data were collected and managed with REDCap (Research Electronic Data Capture) and hosted at Fondazione Policlinico Universitario A. Gemelli IRCCS. All patient information was pseudonymized, and a unique alphanumeric identifier was assigned to each case to ensure secure data linkage.

### 2.3. STAS and IASLC Pattern Evaluation on Surgical Specimens

**Figure 1:**
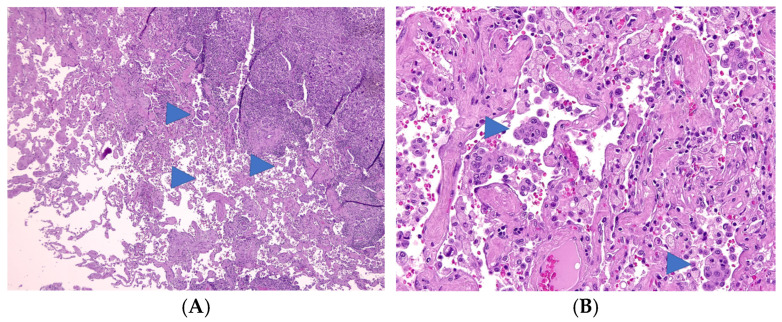
(**A**) Spread through air spaces: Fragments of neoplastic tissue (arrowheads) migrate beyond the edge of the tumor, into adjacent alveoli (H&E, original magnification). (**B**) STAS 3: Several nests of adenocarcinoma are floating freely within alveolar spaces (arrowheads), together with alveolar macrophages. Alveolar septa are thickened by fibrosis, as often observed in smokers (H&E, original magnification 200×).

STAS is defined as the presence of tumor cells within the air spaces beyond the edge of the primary tumor, as previously documented in the literature [11] (Figure 1A,B).

Tumor cells were considered STAS if they were present continuously in the air spaces from the tumor edge, and individual isolated tumor cells or rare tumor clusters were found far away from the tumor without spreading [12]. STAS detection was finally performed on 162 NSCLC patients according to the criteria described below (see Consort Diagram—Supplementary Figure S1). STAS expression was properly evaluated in all anatomical lung resections (137 cases) and non-anatomical ones (i.e., wedge resections) (25 cases), while this factor was not evaluated in biopsies performed in other organs or small lung biopsies, as recommended by the IASLC definition of STAS detection [11].

As reported by Yu and coworkers [13], a false-positive STAS evaluation included: (I) random distribution of tumor cells with irregular edges at the tissue section margins or outside the plane; (II) lack of continuous spread process from the main tumor; (III) serrated edges of tumor cell clusters; (IV) linear bands of cells detached from the alveolar walls; (V) artifacts caused by sectioning blades. We did not perform a further sub-classification according to the “grade” of STAS (single cells, cell nests, micropapillary components) because this has not been largely validated to date.

Similarly, IASLC pattern evaluation was conducted according to the criteria reported in [14]. In cases presenting with more different patterns, the predominant one was reported. Adenocarcinoma grade was not derived in our series, and only the predominant pattern was accounted for.

Expert lung pathologists led the evaluations for STAS and IASLC patterns according to the above-mentioned criteria. Usually, these evaluations were performed by two distinct pathologists who were unaware of the clinical stage and molecular analysis and a consensus was reached in cases of disagreement.

### 2.4. Molecular Analysis

All patients enrolled in the present study were tested via NGS analysis regardless of histology and stage. Hematoxylin and eosin-stained sections were independently reviewed by experienced pathologists to select tumor areas containing a minimum of 20% neoplastic cells. Genomic DNA was isolated from formalin-fixed, paraffin-embedded (FFPE) specimens using the AllPrep^®^ DNA/RNA FFPE Kit (QIAGEN^®^) (Germantown, MD, USA), following the manufacturer’s instructions.

Comprehensive genomic profiling (CGP) was carried out using the TruSight Oncology 500 High Throughput (TSO500HT) panel (Illumina, Inc., San Diego, CA, USA), which allows for the identification of single-nucleotide variants (SNVs), insertions and deletions (indels), and copy number variations (CNVs) across 523 cancer-associated genes. The test is validated for the detection of CNAs, specifically gene amplifications (gain), in 59 genes included in the panel, with a detection limit of 2.2× fold. The TSO500HT is able to identify known and novel fusions in 55 genes of the panel, as well as splicing variants in 3 genes.

The assay also provides evaluation of tumor mutational burden (TMB) and microsatellite instability (MSI) status.

For MSI status, the TSO500HT analyzes 130 homopolymeric sites to calculate an accurate quantitative score. A cutoff of 20% is used to define MSI status (stable < 20%; unstable > 20%). A TMB < 5 mut/Mb is considered low, between 5 and 10 medium, and >10 high. DNA and RNA secondary data analysis are performed using the default parameters on the local TSO500 v2.2 application of Illumina software and within the Clinical Genomics Workspace software platform by PierianDx, TSO500, DRAGEN solid v2.5. The minimum coverage accepted for variant calling is 100× over 90% of the sequenced regions and at least 250× over hotspot regions. Variants with VAF < 5% are identified and called if they have adequate sequencing coverage (>100×) and are reported if present in genes relevant to the clinical question. Copy-number fold change was reported according to the tumor fraction estimation. Only fusion-calling supported by a minimum of 20 fusion-supporting unique reads was reported (DNA quality requirements: >3.5 ng/µL, RNA quality requirements: >10 ng/µL; DV200 > 40%).

Library preparation and next-generation sequencing (NGS) procedures were performed in accordance with the manufacturer’s recommendations and previously published protocols.

Concerning PDL1 analysis, it was performed on formalin-fixed, paraffin-embedded tissue using immunohistochemical detection with the PD-L1 IHC 22C3 pharmDx kit (Agilent Technologies, Inc., Santa Clara, CA, USA) (Dako, REF: SK006), employing the mouse anti-PD-L1 monoclonal antibody (clone 22C3) and the EnVision FLEX visualization system on the Autostainer Link 48 platform (Dako). PD-L1 expression was determined using the Tumor Proportion Score (TPS), defined as the percentage of viable tumor cells showing partial or complete membrane staining. TPS categories are: TPS < 1; 1 ≤ TPS < 50; TPS ≥ 50.

Bioinformatic analysis was conducted using the VELsera Clinical Genomics Workspace (CGW_v8.1.0.3), integrating evidence from curated molecular databases, established clinical guidelines, FDA-approved treatment indications, and currently active clinical trials. Variants were categorized according to the tier-based classification system developed by the Association for Molecular Pathology, the American Society of Clinical Oncology, and the College of American Pathologists [15].

### 2.5. Statistical Analysis

All statistical computations were conducted using R software (version 4.3). Baseline characteristics of the study population were described through summary statistics: continuous variables, such as age and tumor mutational burden (TMB), were reported as medians along with their ranges, whereas categorical variables—including sex, smoking history, clinical stage, histological subtype, IASLC adenocarcinoma patterns, and PD-L1 expression—were presented as counts and percentages.

Comparisons of clinicopathological characteristics between disease stages (early/locally advanced versus advanced) were performed using the Wilcoxon rank-sum test for continuous variables and Fisher’s exact test for categorical variables. The same statistical approach was applied to examine the distribution of PD-L1 expression categories (<1%, 1–49%, ≥50%) and the presence of STAS in relation to clinicopathological parameters, both in the overall cohort and within stage-stratified subgroups.

Genomic data were processed and analyzed using the maftools R package v3.0. For selected biomarkers and variants classified as Tier I–II, the frequency of genomic alterations—including single-nucleotide variants (SNVs), insertions/deletions (indels), and copy number alterations (CNAs)—was calculated for the entire cohort and subsequently compared according to disease stage. Fisher’s exact test was employed to assess overall and stage-specific molecular enrichment, as well as associations between mutation status (e.g., TP53, KRAS, EGFR) and adenocarcinoma growth patterns. Considering the exploratory design of the study, baseline clinicopathological associations were evaluated using nominal two-tailed *p*-values. For high-dimensional genomic comparisons, Fisher’s exact test was initially employed. To rigorously account for multiple hypothesis testing and limit false-positive discoveries within these genomic panels, False Discovery Rate (FDR)-adjusted q-values were subsequently calculated using the Benjamini–Hochberg procedure. Statistical significance was defined as a *p*-value or q-value < 0.05.

## 3. Results

### 3.1. Patient Cohort

A total of 271 patients were included in the study, with an equal sex distribution (49.8% female and 50.2% male) (Table 1). At diagnosis, nearly half of the cohort presented with advanced disease (49.5% stage IV), while 35.8% had early-stage tumors and 14.8% had locally advanced disease. The median age was 69 years (range 28–87).

Histopathological characterization revealed adenocarcinoma as the predominant histotype (89.7%), followed by squamous cell carcinoma (7.3%), with neuroendocrine and mixed tumors accounting for less than 3%. The median tumor mutational burden (TMB) was 6.28 mutations/Mb (range 0–94.3), with low, medium, and high TMB observed in 42.0%, 31.5%, and 26.5% of cases, respectively. Among the 140 adenocarcinoma cases with available subtype information, the most represented growth patterns were acinar (34.3%), solid (25.0%), papillary (15.7%), and mucinous (12.1%), while lepidic (3.6%), micropapillary (7.1%), adenocarcinoma in situ (0.7%), colloid/fetal/enteric (0.7%), and complex/cribriform glandular patterns (0.7%) were less common. According to the above-mentioned criteria, STAS was evaluable in 162 cases and was detected in 17.9% of tumors.

**Figure 2:**
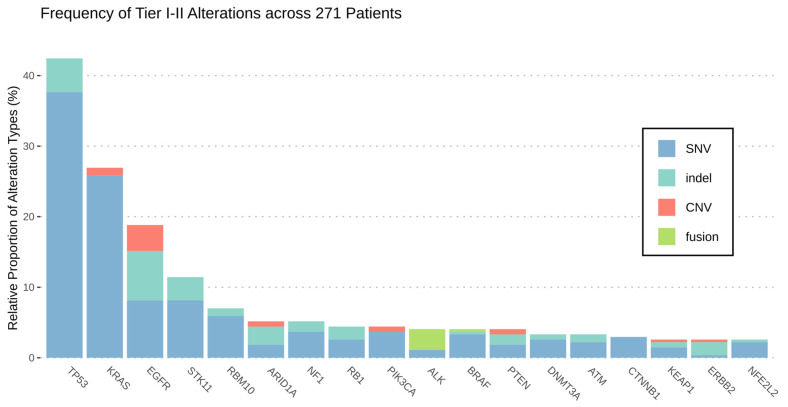
Genomic landscape of somatic alterations (Tier I-II) in the study cohort (N = 271). This stacked bar chart presents the frequency of the most prevalent mutated genes, specifically displaying all genes altered in at least 2% of the patient population. The distinct colors within each bar delineate the specific types of genomic alterations driving these frequencies, including single-nucleotide variants (SNVs), insertions/deletions (Indels), copy number alterations (CNAs), and RNA fusions.

The overall distribution of the most frequently mutated genes is reported in Figure 2.

### 3.2. STAS Distribution in the Whole Cohort and Its Inter-Relationship with Clinicopathological Features

A total of 162 patients were considered for STAS analysis, comprising 133 cases that were negative for STAS and 29 positive (Table 2). No significant differences were observed between the two groups in terms of sex distribution (*p* = 0.8385), with females accounting for 48.1% of STAS-negative cases and 44.8% of STAS-positive cases. The median age at diagnosis was 69 years in both groups (STAS-negative: 28–85; STAS-positive: 50–86; *p* = 0.815).

Tumor stage showed a non-significant trend toward a higher proportion of locally advanced and advanced disease in the STAS-positive group (*p* = 0.0617). Early-stage tumors were more frequently STAS-negative (63.9%) compared to STAS-positive (41.4%), whereas locally advanced disease was more commonly STAS-positive (37.9% vs. 21.1%).

Regarding histotype, adenocarcinoma was the predominant subtype in both groups and accounted for all STAS-positive cases (100%), while other histotypes (atypical carcinoid, NEC, and squamous cell carcinoma) were observed only in the STAS-negative group (*p* = 0.0799).

No statistically significant differences were found in TMB grade distribution (*p* = 0.5165), smoking status (*p* = 0.1876), or PD-L1 expression levels (*p* = 0.5341) between the STAS-negative and STAS-positive groups.

In contrast, adenocarcinoma growth pattern was significantly associated with STAS expression (*p* = 2.50 × 10^−6^). STAS tumors were enriched for micropapillary (25% vs. 1.1%), mucinous (21.4% vs. 6.8%), and papillary (25% vs. 14.8%) patterns, whereas the acinar pattern was markedly less frequent in STAS-positive compared with STAS-negative tumors (10.7% vs. 51.1%).

Somatic mutational profiles (copy number, single-nucleotide variation and DNA) were comparable between STAS-negative (*n* = 133) and STAS-positive (*n* = 29) tumors. The most frequently altered genes were TP53, KRAS, EGFR, and STK11, with no significant differences between groups (all *p* > 0.05). TP53 mutations were observed in 36.1% of STAS-negative and 24.1% of STAS-positive cases, while KRAS mutations occurred in 24.1% and 31.0%, respectively.

No other recurrent gene alteration showed a statistically significant association with STAS status. A non-significant trend toward higher mutation frequency in STAS tumors was noted for TET2, CTNNB1, SETD2, and FANCA (all *p* > 0.05).

Considering somatic mutational profiles for RNA instead, ROS1 alterations were absent in STAS-negative tumors but detected in 6.9% of STAS-positive cases, representing a statistically significant difference (*p* = 0.0311). Overall, with the exception of ROS1, no significant differences in the distribution of the analyzed gene alterations were observed between the two groups (Table 3).

### 3.3. STAS Distribution in the Early/Locally Advanced Stages and Its Inter-Relationship with Clinicopathological Features

The early–locally advanced cohort comprised 136 patients (Table 4). No significant differences were observed with respect to sex distribution (*p* = 0.8199), with females representing 47.79% of STAS-negative cases and 43.48% of STAS-positive cases. Median age at diagnosis was comparable between groups (70 years [range 28–85] in STAS-negative vs. 69 years [range 50–86] in STAS-positive; *p* = 0.5809).

A statistically significant association emerged for disease stage (*p* = 0.0409), as STAS-positive tumors were more frequently locally advanced (47.83% vs. 24.78%), whereas STAS-negative cases were predominantly early stage (75.22% vs. 52.17%). Histotype distribution did not significantly differ between groups (*p* = 0.0966), although all STAS-positive tumors were adenocarcinomas.

No significant associations were identified between STAS status and tumor mutational burden (TMB) categories (*p* = 0.4279), smoking history (*p* = 0.2044), or PD-L1 expression levels (*p* = 0.2733).

In contrast, a highly significant difference was observed in adenocarcinoma growth patterns (*p* = 6.30 × 10^−5^). The acinar pattern was predominant in STAS-negative tumors (55.13% vs. 13.64%), whereas the micropapillary pattern was markedly enriched in STAS-positive cases (27.27% vs. 1.28%). Papillary growth was also more frequent among STAS-positive tumors (31.82% vs. 16.67%).

Considering somatic mutational profiles for RNA for this subgroup, ROS1 alterations were absent in STAS-negative tumors but detected in 8.7% of STAS-positive cases (2 out of 23), representing a statistically significant difference (*p* = 0.0276). Overall, with the exception of ROS1, no significant differences in the distribution of the analyzed gene alterations were observed between the two groups for the early–locally advanced stage cohort (Table 5).

### 3.4. STAS Distribution in the Advanced Stages and Its Inter-Relationship with Clinicopathological Features

Clinicopathological characteristics were compared between STAS-negative and positive cases, within the advanced stage subgroup, which comprises 26 patients. Sex distribution was identical in the two groups (50% female and 50% male in both). The median age at diagnosis was comparable, with 68.5 years (range 48–79) in the STAS-negative group and 68 years (range 56–74) in the STAS-positive group.

Histologically, adenocarcinoma was the predominant subtype in both cohorts (95% in STAS-negative vs. 100% in STAS-positive), with a single squamous cell carcinoma observed only in the STAS-negative group. Tumor mutational burden (TMB) categories were evenly distributed.

The solid pattern was the most frequent subtype in STAS-negative tumors (70%), whereas STAS-positive cases more commonly exhibited mucinous features (50%). Notably, a micropapillary pattern was identified exclusively in the STAS-positive group (16.67%), while acinar and lepidic patterns were observed only in STAS-negative tumors (Table 6).

The most frequently altered gene in the STAS-negative group was TP53 (40%), followed by STK11 (25%), while KRAS and EGFR mutations were each detected in 15% of STAS positive cases.

## 4. Discussion

The comprehensive examination of our real-world lung cancer patients in this study revealed significant insights into the interesting interplay between STAS, pathologic features, and gene mutations.

Considering the important prognostic role of STAS in lung cancer, early identification and prediction may be useful in guiding surgical methods and improving prognosis. If STAS proves to have real clinical relevance and supports the choice of lobectomy rather than sublobar resection, the identification of an association with clinicopathological features would represent an important component in the management of patients with lung adenocarcinoma, as it could lead surgeons to opt immediately for lobectomy instead of a limited resection.

In our analysis, we observed that STAS presence trends toward significant association with advanced stages and is significantly associated with adenocarcinoma histology; concerning adenocarcinoma subtypes, STAS presence is strongly associated with papillary and micropapillary histotypes, while acinar adenocarcinoma was usually found in STAS-negative cases. No other statistically significant associations were found with clinicopathological features, PDL1 levels, and tumor mutational burden.

However, as reported below, considering a bias of selection related to the possibility of assessing STAS in surgical specimens only (26 out of 134 advanced patients), a direct comparison between the advanced and early–locally advanced groups was not entirely methodologically correct.

Concerning the mutational profile in the whole cohort and taking into account the early/locally advanced group, somatic mutations of ROS1 in the RNA were more frequent in patients with STAS expression.

Uruga et al. [16] studied 208 cases of early-stage lung adenocarcinoma and found a significant association between higher STAS, solid-predominant histology and tumor size ≥ 10 mm.

Micropapillary-predominant, high-stage, node-positive, and metastasized adenocarcinomas were significantly associated with the presence of STAS in 569 resected pulmonary adenocarcinomas of any stage, as reported by Warth et al. [17].

Male sex and cigarette smoking were related to the presence of STAS in 318 stage I adenocarcinomas, as reported by Shiono and Yanagawa [18]; however, these correlations were not found in our analysis, both in the whole cohort and when considering separate early–locally advanced stage and advanced stage groups.

Other studies also reported a significant association between STAS and different parameters, including micropapillary growth pattern, nodal involvement, and a consequent higher stage [19,20,21].

Although we did not investigate the long-term survival in this study, our findings may potentially suggest that STAS presence is theoretically associated with a worse prognosis, as it has been shown to be associated with papillary and micropapillary adenocarcinoma. It is indeed well known that papillary and micropapillary lung adenocarcinoma have a shorter survival than the acinar ones [22], and of course, patients with more advanced stages have worse outcomes compared to earlier ones. It is less clear if STAS itself may have some direct influence on the prognosis or if this association depends on the interrelationship between STAS and other prognostic factors.

Several studies have found that STAS presence was negatively associated with EGFR mutation and positively correlated with BRAF and KRAS mutations, while others have reported no association with EGFR [23] or KRAS [24] status. STAS has also been found more frequently in tumors with ROS1 rearrangements [25]. Results of the present study show that ROS1 rearrangement was the most frequent alteration in STAS-positive adenocarcinoma, in accordance with the findings of Tian and coworkers [26] and Jia and coworkers [27].

As Jia and colleagues report, the association between STAS and molecular characteristics has yet to be clearly explicated. One possible explanation for the different frequencies of STAS based on different driver gene alterations could be that STAS is more frequently observed in poorly differentiated tumors, including those with papillary and micropapillary patterns [28], and ALK or ROS1 rearrangements mainly exist in adenocarcinoma with solid/papillary patterns [29].

Limitations of this study include some center-specific biases that, despite being limited by a strict protocol of data management, are substantially unavoidable even in the context of a prospective observational trial.

Moreover, the lack of external validation remarkably reduces the generalizability of our results.

The small number of STAS-positive patients and the small number of advanced stage patients represent other limitations that we need to declare. Indeed, the group of the advanced stage patients contains only 26 out of the 134 total advanced patients, due to the pre-selection of patients (STAS was properly evaluated only on surgical specimens). For this reason, a direct comparison between the advanced and early–locally advanced groups was not entirely methodologically correct. Another limitation was the lack of survival results (not yet mature at the time of writing the present manuscript). Retrospective examination of pathological sections was not conducted, and interobserver agreement was not assessed.

## 5. Conclusions

The present study comprehensively analyzes clinicopathological and genetic features according to STAS expression in lung cancer patients. The poorly differentiated histological subtypes were shown to be related to STAS, especially in advanced stages, despite having a small cohort. No definitive conclusions can be drawn about the relation to genomic mutations.

The result of this research is expected to provide direction for further investigation on the dominating factors and molecular mechanisms of STAS presence.

## Figures and Tables

**Table 1:** Clinicopathological features.

	N = 271	%
Sex		
Female	135/271	49.82%
Male	136/271	50.18%
Stage		
Early (stage I)	97/271	35.79%
Locally Advanced (stage II–III)	40/271	14.76%
Advanced (stage IV)	134/271	49.45%
Kind of histology acquisition		
Surgery (anatomical lung resection)	137/271	50.5%
Surgery (non-anatomical lung resection)	25/271	9.2%
Biopsy (from primary or metastatic site)	109/271	40.3%
Age at diagnosis		
Median (min–max)	69 (28–87)	
Smoker		
Yes	75/268	27.99%
Ex-smoker	126/268	47.01%
No	67/268	25%
PD-L1		
<1%	78/229	34.06%
1–49%	66/229	28.82%
>50%	85/229	37.12%
Histotype		
Adenocarcinoma	234/271	86.3%
Adenosquamous	2/271	0.74%
NEC/NSCLC	6/271	2.2%
Squamous cell carcinoma	19/271	7.1%
NOS	10/271	3.66%
STAS expression		
No	133/162	82.1%
Yes	29/162	17.9%
TMB		
Median (min–max)	6.28 (0–94.3)	
TMB grade		
Low	92/219	42.01%
Medium	69/219	31.51%
High	58/219	26.48%
MSI grade		
Stable	220/222	99.1%
Unstable	2/222	0.9%
ADC subtype growth pattern		
Acinar pattern	48/140	34.29%
Adenocarcinoma in situ	1/140	0.71%
Colloid/fetal/enteric pattern	1/140	0.71%
Complex/cribriform glandular pattern	1/140	0.71%
Lepidic pattern	5/140	3.57%
Micropapillary pattern	10/140	7.14%
Mucinous pattern	17/140	12.14%
Papillary pattern	22/140	15.71%
Solid pattern	35/140	25%

**Table 2:** Clinicopathological features in the STAS-analyzed cohort.

Clinical Features	STAS-Negative	STAS-Positive	*p*-Value
Sex			0.8385
Female	64/133 (48.12%)	13/29 (44.83%)	
Male	69/133 (51.88%)	16/29 (55.17%)	
Age at diagnosis			0.815
Median (min–max)	69 (28–85)	69 (50–86)	
Stage			0.0617
Early	85/133 (63.91%)	12/29 (41.38%)	
Locally Advanced	28/133 (21.05%)	11/29 (37.93%)	
Advanced	20/133 (15.04%)	6/29 (20.69%)	
Histotype			0.0799
Adenocarcinoma	108/133 (81–2%)	29/29 (100%)	
Atypical carcinoid	2/133 (1.51%)	0/29 (0%)	
NEC	4/133 (3.01%)	0/29 (0%)	
Squamous cell carcinoma	19/133(14.28%)	0/29 (0%)	
TMB grade			0.5165
Low	43/107 (40.19%)	9/19 (47.37%)	
Medium	37/107 (34.58%)	4/19 (21.05%)	
High	27/107 (25.23%)	6/19 (31.58%)	
Smoker			0.1876
Yes	40/132 (30.3%)	5/29 (17.24%)	
Ex-smoker	68/132 (51.52%)	15/29 (51.72%)	
No	24/132 (18.18%)	9/29 (31.03%)	
PDL1 expression			0.5341
<1%	53/106 (50%)	10/24 (41.67%)	
>50%	27/106 (25.47%)	9/24 (37.5%)	
1–49%	26/106 (24.53%)	5/24 (20.83%)	
ADC subtype growth pattern			2.50 × 10^−6^
Acinar pattern	45/88 (51.14%)	3/28 (10.71%)	
Lepidic pattern	5/88 (5.68%)	0/28 (0%)	
Micropapillary pattern	1/88 (1.14%)	7/28 (25%)	
Mucinous pattern	6/88 (6.82%)	6/28 (21.43%)	
Papillary pattern	13/88 (14.77%)	7/28 (25%)	
Solid pattern	18/88 (20.45%)	5/28 (17.86%)	

**Table 3:** Distribution of RNA-level somatic alterations across the STAS-analyzed cohort. The table details the frequency of gene fusions and RNA-level events stratified by STAS status (Negative vs. Positive). To rigorously account for multiple hypothesis testing across the six evaluated genes, False Discovery Rate (FDR)-adjusted q-values were used.

Gene	STAS-Negative	STAS-Positive	*p*-Value	q-Value
ALK	4/133 (3.01%)	1/29 (3.45%)	1	1
RET	3/133 (2.26%)	0/29 (0%)	1	1
EML4	0/133 (0%)	1/29 (3.45%)	0.1790	0.5370
ROS1	0/133 (0%)	2/29 (6.9%)	0.0311	0.1866
BRAF	1/133 (0.75%)	0/29 (0%)	1	1
NRG1	1/133 (0.75%)	0/29 (0%)	1	1

**Table 4:** Clinicopathological features in the early and locally advanced stage cohort.

Clinical Features	STAS-Negative	STAS-Positive	*p*-Value
Sex			0.8199
Female	54/113 (47.79%)	10/23 (43.48%)	
Male	59/113 (52.21%)	13/23 (56.52%)	
Age at diagnosis			0.5809
Median (min–max)	70 (28–85)	69 (50–86)	
Stage			0.0409
Early	85/113 (75.22%)	12/23 (52.17%)	
Locally Advanced	28/113 (24.78%)	11/23 (47.83%)	
Histotype			0.0966
Adenocarcinoma	84/108 (77.78%)	23/23 (100%)	
Atypical carcinoid	2/108 (1.85%)	0/23 (0%)	
NEC	4/108 (3.7%)	0/23 (0%)	
Squamous cell carcinoma	18/108 (16.67%)	0/23 (0%)	
TMB grade			0.4279
Low	37/91 (40.66%)	8/16 (50%)	
Medium	32/91 (35.16%)	3/16 (18.75%)	
High	22/91 (24.18%)	5/16 (31.25%)	
Smoker			0.2044
Yes	34/113 (30.09%)	3/23 (13.04%)	
Ex-smoker	59/113 (52.21%)	14/23 (60.87%)	
No	20/113 (17.7%)	6/23 (26.09%)	
PDL1 expression			0.2733
<1%	48/88 (54.55%)	7/18 (38.89%)	
>50%	21/88 (23.86%)	8/18 (44.44%)	
1–49%	19/88 (21.59%)	3/18 (16.67%)	
ADC subtype growth pattern			6.30 × 10^−5^
Acinar pattern	43/78 (55.13%)	3/22 (13.64%)	
Lepidic pattern	4/78 (5.13%)	0/22 (0%)	
Micropapillary pattern	1/78 (1.28%)	6/22 (27.27%)	
Mucinous pattern	6/78 (7.69%)	3/22 (13.64%)	
Papillary pattern	13/78 (16.67%)	7/22 (31.82%)	
Solid pattern	11/78 (14.1%)	3/22 (13.64%)	

**Table 5:** Distribution of RNA-level somatic alterations in the early and locally advanced stage cohort. The table details the frequency of gene fusions and RNA-level events stratified by STAS status (Negative vs. Positive). To rigorously account for multiple hypothesis testing across the six evaluated genes, False Discovery Rate (FDR)-adjusted q-values were used.

Gene	STAS-Negative	STAS-Positive	*p*-Value	q-Value
ALK	2/113 (1.77%)	1/23 (4.35%)	0.429	1
RET	2/113 (1.77%)	0/23 (0%)	1	1
ROS1	0/113 (0%)	2/23 (8.7%)	0.0276	0.1380
BRAF	1/113 (0.88%)	0/23 (0%)	1	1
NRG1	1/113 (0.88%)	0/23 (0%)	1	1

**Table 6:** Clinicopathological features in the advanced stage cohort.

Clinical Features	STAS-Negative	STAS-Positive
Sex		
Female	10/20 (50%)	3/6 (50%)
Male	10/20 (50%)	3/6 (50%)
Age at Diagnosis		
Median (min–max)	68.5 (48–79)	68 (56–74)
Stage		
Advanced	20/20 (100%)	6/6 (100%)
Histotype		
Adenocarcinoma	19/20 (95%)	6/6 (100%)
Squamous cell carcinoma	1/20 (5%)	0/6 (0%)
TMB grade		
Low	6/16 (37.5%)	1/3 (33.33%)
Medium	5/16 (31.25%)	1/3 (33.33%)
High	5/16 (31.25%)	1/3 (33.33%)
Smoker		
Yes	6/19 (31.58%)	2/6 (33.33%)
Ex-smoker	9/19 (47.37%)	1/6 (16.67%)
No	4/19 (21.05%)	3/6 (50%)
PDL1 expression		
<1%	5/18 (27.78%)	3/6 (50%)
>50%	6/18 (33.33%)	1/6 (16.67%)
1–49%	7/18 (38.89%)	2/6 (33.33%)
ADC subtype growth pattern		
Acinar pattern	2/10 (20%)	0/6 (0%)
Lepidic pattern	1/10 (10%)	0/6 (0%)
Micropapillary pattern	0/10 (0%)	1/6 (16.67%)
Mucinous pattern	0/10 (0%)	3/6 (50%)
Solid pattern	7/10 (70%)	2/6 (33.33%)

## Data Availability

The original contributions presented in this study are included in the article and Supplementary Materials. Further inquiries can be directed to the corresponding author.

## Supplementary Materials

The following supporting information can be downloaded at: https://www.mdpi.com/article/10.3390/genes17060677/s1, Figure S1: Consort diagram.
