# Supplementary material for: LANTERN 2: Association Between Gene Molecular Profile and STAS in Lung Adenocarcinoma: A Comparative Analysis in a Prospective Real-World Population
Source: Genes (Basel). 2026 Jun 9;17(6):677. doi: 10.3390/genes17060677 (PMC13300064; doi:10.3390/genes17060677)
Supplement: Supplementary file 1 [file genes-17-00677-s001.zip › genes-4295326-supplementary.pptx]

## Slide 1
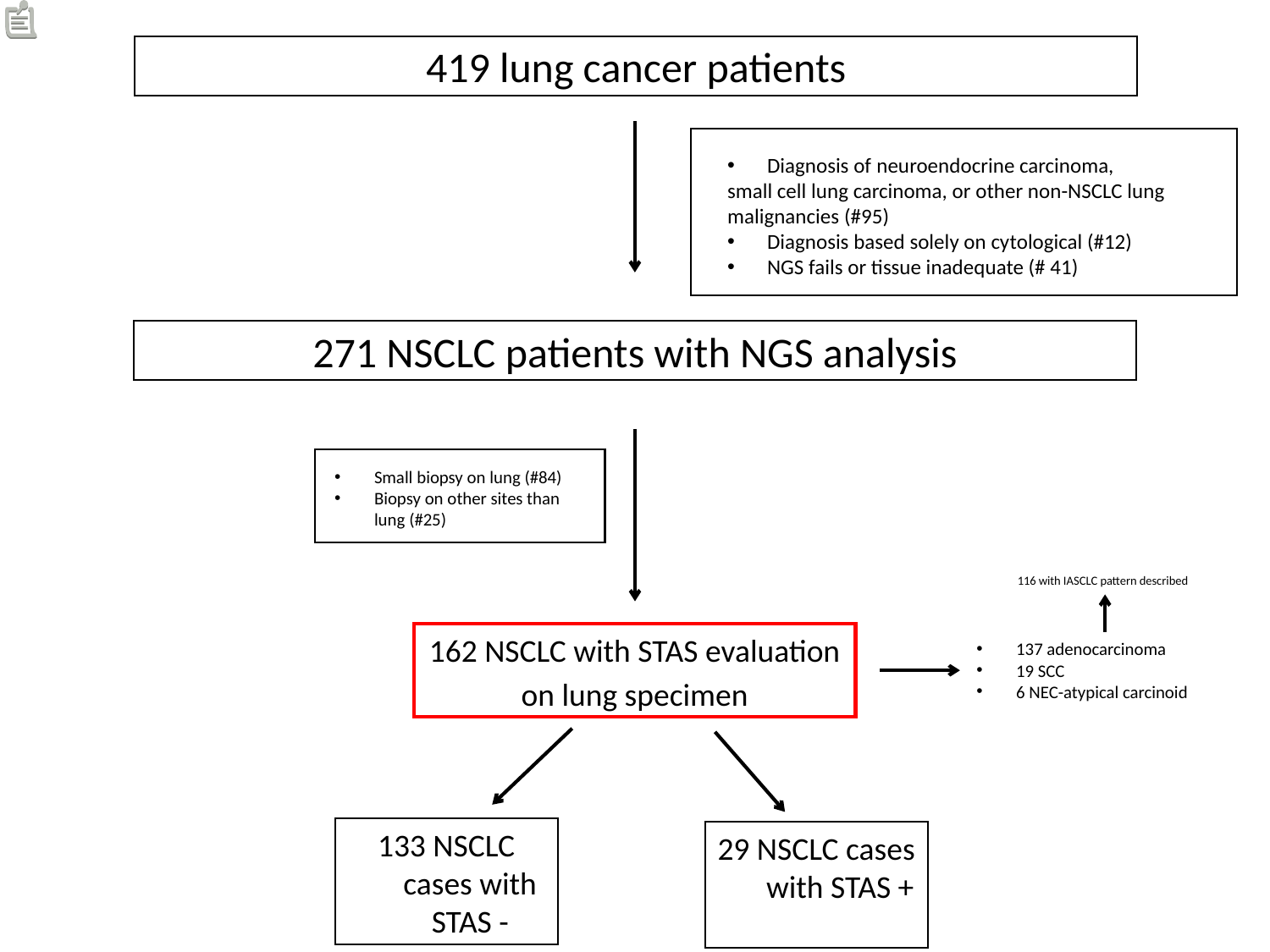

419 lung cancer patients
Diagnosis of neuroendocrine carcinoma,
small cell lung carcinoma, or other non-NSCLC lung malignancies (#95)
Diagnosis based solely on cytological (#12)
NGS fails or tissue inadequate (# 41)
271 NSCLC patients with NGS analysis
Small biopsy on lung (#84)
Biopsy on other sites than lung (#25)
116 with IASCLC pattern described
162 NSCLC with STAS evaluation
on lung specimen
137 adenocarcinoma
19 SCC
6 NEC-atypical carcinoid
133 NSCLC cases with STAS -
29 NSCLC cases with STAS +
